# In vitro activity of aztreonam–avibactam against Enterobacterales isolates collected in Latin America, Africa/Middle East, Asia, and Eurasia for the ATLAS Global Surveillance Program in 2019–2021

**DOI:** 10.1007/s10096-023-04645-2

**Published:** 2023-08-01

**Authors:** Mark G. Wise, James A. Karlowsky, Naglaa Mohamed, Shweta Kamat, Daniel F. Sahm

**Affiliations:** 1 IHMA, 2122 Palmer Drive, Schaumburg, IL 60173 USA; 2 Department of Medical Microbiology and Infectious Diseases, Max Rady College of Medicine, University of Manitoba, Winnipeg, MB Canada; 3 Pfizer Inc., New York, NY USA; 4 Pfizer, Mumbai India

**Keywords:** ATLAS, Aztreonam–avibactam, Emerging markets, Enterobacterales, Surveillance, Metallo-β-lactamase

## Abstract

This study aimed to report reference method antimicrobial susceptibility results for 24,937 recent (2019–2021) clinical isolates of Enterobacterales from 27 countries in Latin America, Eurasia, Africa/Middle East, and Asia with a focus on the investigational combination aztreonam–avibactam against metallo-β-lactamase (MBL) isolates. Antimicrobial susceptibility testing was performed by the CLSI broth microdilution methodology. Minimum inhibitory concentrations (MICs) were interpreted using the CLSI (2022) breakpoints for all agents except aztreonam–avibactam (provisional pharmacokinetic/pharmacodynamic susceptible breakpoint, ≤ 8 mg/L) and tigecycline (US-FDA). Molecular testing for β-lactamase genes was performed on isolates with meropenem MICs ≥ 2 mg/L, ceftazidime–avibactam MICs ≥ 16 mg/L, and/or aztreonam–avibactam MICs ≥ 16 mg/L, and 50% of isolates of *Escherichia coli*, *Klebsiella pneumoniae*, *Klebsiella oxytoca*, *Klebsiella variicola*, and *Proteus mirabilis* testing with ceftazidime and/or aztreonam MICs ≥ 2 mg/L. Aztreonam–avibactam inhibited 99.8% of all Enterobacterales at ≤ 8 mg/L (MIC_90_, 0.25 mg/L) and maintained activity against phenotypically resistant subsets of multidrug-resistant (MDR) (99.5% susceptible), extensively drug-resistant (XDR) (98.7%), and carbapenem-resistant Enterobacterales (CRE) (99.1%) isolates. At ≤ 8 mg/L, aztreonam–avibactam inhibited 100%, 99.6%, 99.6%, and 98.8% of KPC-, OXA-48-like-, ESBL-, and MBL-carrying isolates, respectively. MBL-positive isolates were most prevalent in India (20.5%), Guatemala (13.8%), and Jordan (13.2%). No differences in the activity of aztreonam–avibactam were observed across the global regions evaluated. At a concentration of ≤ 8 mg/L, aztreonam–avibactam inhibited almost all Enterobacterales collected from developing countries, including MBL-producing isolates. The widespread dissemination of MBLs among Enterobacterales highlights the unmet need for new agents such as aztreonam–avibactam for the treatment of CRE infections.

**Supplementary Information:**

The online version contains supplementary material available at 10.1007/s10096-023-04645-2.

## Introduction

Bacterial resistance to antimicrobial agents is a well-documented threat to modern medicine. Among Gram-negative bacteria, the spread of acquired MBLs is particularly concerning, as they have the ability to hydrolyze all currently available β-lactam antibiotics, except for the monobactams and cefiderocol [1]. Recently approved β-lactam/β-lactamase inhibitor combinations, such as ceftazidime–avibactam, have proven activity against multidrug-resistant (MDR) bacterial infections [2]; however, ceftazidime–avibactam, imipenem–relebactam, and meropenem–vaborbactam are not active against metallo-β-lactamase (MBL)-harboring Gram-negative bacilli in vitro, leaving a gap in the current antimicrobial armamentarium and highlighting the need for new agents to treat infections caused by these pathogens [3]. Aztreonam–avibactam is in late development for use against infections caused by carbapenem-resistant Enterobacterales (CRE), including isolates carrying MBLs [4]. Aztreonam is refractory to hydrolysis by MBLs but is inactivated by ESBLs and other class A β-lactamases including KPCs and plasmid-mediated or stably derepressed chromosomally encoded class C (AmpC) β-lactamases. The β-lactamase inhibitor, avibactam, inhibits the activities of class A, C, and certain class D (including OXA-48-like) β-lactamases that are frequently co-carried with MBLs. As such, aztreonam–avibactam, if approved, would represent an attractive therapeutic option for use against MBL-harboring Enterobacterales regardless of whether or not they co-carried serine β-lactamase(s).

The Antimicrobial Testing Leadership and Surveillance (ATLAS) Global Surveillance Program offers a means to annually monitor the in vitro activity of antimicrobials, including aztreonam–avibactam, against clinical isolates of Gram-negative bacilli collected worldwide. Isolates associated with bloodstream, intra-abdominal, respiratory tract, skin and soft tissue, and urinary tract infections are included in the program. The current study focused on the in vitro activity of aztreonam–avibactam and comparators against Enterobacterales collected in 27 countries in Latin America, Eurasia, the Africa/Middle East region, and Asia in 2019–2021 for which current, robust, and country-specific in vitro aztreonam–avibactam data is not widely available.

## Materials and methods

### Bacterial isolates

Isolates of Enterobacterales (*n* = 24,937) included in the present study were collected as a part of the ATLAS Global Surveillance Program from 27 countries worldwide between 2019 and 2021. For analysis, countries were grouped into four geographic regions as follows: the Africa/Middle East region included Cameroon, Ivory Coast, Jordan, Kuwait, Morocco, Nigeria, Qatar, Saudi Arabia, and South Africa; Asia included Hong Kong, India, Malaysia, the Philippines, Taiwan, and Thailand; Eurasia included Russia and Turkey; and Latin America included Argentina, Brazil, Chile, Colombia, Costa Rica, Dominican Republic, Guatemala, Mexico, Panama, and Venezuela (Supplemental Table S1). Medical centers in each country participated in the ATLAS program in all 3 years with the exception of Cameroon and Ivory Coast which participated only in 2020 and 2021, and Russia, from which isolates were only available in 2019. Organisms in the present study were isolated from the bloodstream (*n* = 5921), intra-abdominal (*n* = 3263), lower respiratory tract (*n* = 4907), skin and soft tissue (*n* = 4400), urinary tract (*n* = 6408), and other unspecified (*n* = 38) infection sources. Isolates collected from ICU patients accounted for 26.7% (6632/24,937) of the total isolates. Bacterial species identities were confirmed by IHMA (Schaumburg, IL, USA) using the MALDI-TOF mass spectrometry (Bruker Daltonics, Billerica, MA, USA).

The ATLAS program requests each participating medical center laboratory to collect annually defined quotas of isolates of selected bacterial species from patients with bloodstream infections, intra-abdominal infections, lower respiratory tract infections, skin and soft tissue infections, and urinary tract infections. It was limited to one isolate per species per patient. All isolates were determined to be clinically significant by participating laboratory algorithms and were collected irrespective of antimicrobial susceptibility profile. The ATLAS program (https://atlas-surveillance.com) is not intended to evaluate the geographic prevalence of bacteria causing specific infection types. As with all large-scale antimicrobial resistance surveillance networks, ATLAS does experience some variation in the number of participating centers in each study year, center constancy between study years, and distribution of centers in each region.

### Antimicrobial susceptibility testing

Antimicrobial susceptibility testing was performed in a central laboratory (IHMA) using the Clinical and Laboratory Standards Institute (CLSI) broth microdilution method [5]. Avibactam was tested at a fixed concentration of 4 mg/L. Minimum inhibitory concentrations (MICs) were interpreted using the 2022 CLSI [6] breakpoints (interpretation via 2022 EUCAST criteria [7] is provided in Supplemental Table S2). United States Food and Drug Administration (FDA) MIC interpretative breakpoints were used for tigecycline [8] in place of CLSI breakpoints which do not exist. Aztreonam–avibactam MICs were interpreted using a previously established provisional pharmacokinetic/pharmacodynamic susceptible breakpoint of ≤ 8 mg/L [9–11].

Isolates were identified as CRE on the basis of imipenem or meropenem MIC values ≥ 4 mg/L, with the exception that imipenem was excluded from CRE screening of Morganellaceae due to its intrinsically elevated MIC values in that family. Isolates were categorized as MDR or XDR according to criteria published in 2012 by the joint European and United States Centers for Disease Control [12], which define an MDR isolate as one that is nonsusceptible to ≥ 1 agent in ≥ 3 antimicrobial classes and an XDR isolate as one that is susceptible to ≤ 2 classes from the agents tested. The antimicrobial classes and specific antimicrobial agent representatives in this analysis were cephalosporins (ceftazidime, cefepime), cephalosporins combined with β-lactamase inhibitor (ceftazidime–avibactam), carbapenems (imipenem, meropenem), fluoroquinolones (levofloxacin), aminoglycosides (gentamicin, amikacin), monobactams (aztreonam), polymyxins (colistin), and glycylcyclines (tigecycline).

### Molecular analysis

Isolates of Enterobacterales that tested with a meropenem MIC of ≥ 2 mg/L (not susceptible by the CLSI breakpoint), a ceftazidime–avibactam MIC of ≥ 16 mg/L (not susceptible by the CLSI breakpoint), and/or an aztreonam–avibactam MIC of ≥ 16 mg/L were screened for the presence of genes encoding serine carbapenemases (KPC, OXA-48-like, and GES), MBLs (NDM, IMP, VIM, SPM, and GIM), ESBLs (SHV, TEM, CTX-M-1 group, CTX-M-2 group, CTX-M-8 group, CTX-M-9 group, CTX-M-25 group, VEB, PER, and GES), and acquired AmpC β-lactamases (ACC, ACT, CMY, DHA, FOX, MIR, and MOX) using multiplex PCR assays, followed by amplification and sequencing of the full-length genes and comparison to publicly available databases, as previously described [13]. Additionally, 50% of meropenem-susceptible (MIC ≤ 1 mg/L) isolates of *Escherichia coli*, *Klebsiella pneumoniae*, *Klebsiella oxytoca*, *Klebsiella variicola*, and *Proteus mirabilis* testing with ceftazidime or aztreonam MICs ≥ 2 mg/L (CLSI ESBL screening criteria) [6] were interrogated for β-lactamase genes following the same procedure. The acquired β-lactamase carriage of isolates testing with aztreonam–avibactam MIC values ≥ 16 mg/L is provided in the “Supplemental Table S3.”

## Results

Table 1 summarizes the in vitro susceptibility testing data for 12 antimicrobial agents against all 24,937 Enterobacterales isolates, collected from 2019 to 2021, from the 27 countries. The growth of 99.8% of isolates from the full collection was inhibited by aztreonam–avibactam at a concentration of ≤ 8 mg/L, the provisional pharmacokinetic/pharmacodynamic susceptible breakpoint [9–11], and the MIC_90_ value was 0.25 mg/L. Only 61 of the 24,937 (0.2%) isolates tested with an aztreonam–avibactam MIC > 8 mg/L, including 38 *E. coli*, seven *K. pneumoniae*, five *P. mirabilis*, three *Providencia rettgeri*, and eight members of other species of Enterobacterales. Tigecycline (96.4% susceptible), ceftazidime–avibactam (93.1%), and amikacin (91.3%) also inhibited > 90% of all isolates at their respective susceptible breakpoints: 86.9% of all isolates were meropenem-susceptible, and aztreonam (59.9% susceptible), cefepime (60.6%), and ceftazidime (59.6%) showed similar activities. Only 54.8% of all Enterobacterales isolates were levofloxacin-susceptible.

**Table 1:** In vitro activity of aztreonam–avibactam and comparator agents against Enterobacterales collected in emerging market countries, 2019–2021

Region^b^	Antimicrobial agent^a^																							
Phenotype/genotype (no. of isolates)	MIC_90_ (mg/L)/% susceptible (%S)																							
	ATM–AVI		ATM		AMK		FEP		CAZ		CZA		CST^c^		GEN		IPM		LVX		MEM		TGC^d^	
	MIC_90_	% ≤ 8 (mg/L^e^)	MIC_90_	%S	MIC_90_	%S	MIC_90_	%S	MIC_90_	%S	MIC_90_	%S	MIC_90_	%S	MIC_90_	%S	MIC_90_	%S	MIC_90_	%S	MIC_90_	%S	MIC_90_	%S
All regions																								
All Enterobacterales (24,937)	0.25	99.8	> 64	59.9	16	91.3	> 32	60.6	> 64	59.6	1	93.1	> 8	81.5	> 16	72.7	8	77.0	> 8	54.8	16	86.9	2	96.4
MDR^f^ (12,192)	0.5	99.5	> 64	18.3	> 64	82.3	> 32	20.0	> 64	18.6	> 64	86.0	> 8	82.7	> 16	45.9	> 8	62.1	> 8	20.8	> 16	73.3	2	93.4
XDR^f^ (2974)	1	98.7	> 64	5.8	> 64	42.1	> 32	1.2	> 64	2.2	> 64	47.6	> 8	77.2	> 16	16.9	> 8	6.0	> 8	2.7	> 16	12.1	4	88.6
CRE^g^ (3289)	1	99.1	> 64	12.4	> 64	50.0	> 32	4.7	> 64	6.6	> 64	49.9	> 8	79.6	> 16	34.2	> 8	0.8	> 8	10.7	> 16	5.4	2	94.4
MBL + (1610)	2	98.8	> 64	16.1	> 64	42.2	> 32	0.4	> 64	0.2	> 64	1.0	> 8	83.4	> 16	28.3	> 8	0.9	> 8	9.6	> 16	1.8	2	93.8
KPC^h^ + (705)	0.5	100	> 64	0.7	64	69.6	> 32	4.4	> 64	10.1	2	99.0	> 8	71.1	> 16	49.6	> 8	1.6	> 8	13.6	> 16	4.0	2	95.0
OXA-48-like^i^ + (831)	0.5	99.6	> 64	8.2	> 64	46.7	> 32	5.2	> 64	7.9	2	98.0	> 8	81.7	> 16	27.8	> 8	8.2	> 8	3.9	> 16	13.8	2	95.8
ESBL^j^ + (3605)	0.25	99.6	> 64	5.7	16	92.7	> 32	4.8	> 64	13.0	1	98.9	1	96.3	> 16	49.2	> 8	21.8	> 8	21.8	0.12	95.0	1	97.2
Africa/Middle East																								
All Enterobacterales (5245)	0.25	99.9	> 64	59.6	8	95.7	> 32	60.0	> 64	59.4	1	94.5	> 8	82.5	> 16	70.7	4	81.0	> 8	56.1	0.5	92.0	2	96.5
MDR (2576)	0.25	99.7	> 64	18.1	16	91.4	> 32	19.1	> 64	18.4	> 64	88.9	> 8	85.2	> 16	41.5	> 8	71.0	> 8	24.2	> 16	83.9	2	93.8
XDR (428)	0.5	98.8	> 64	6.3	> 64	63.6	> 32	2.8	> 64	1.2	> 64	37.9	> 8	80.8	> 16	23.8	> 8	4.7	> 8	5.8	> 16	18.9	4	86.7
CRE (446)	0.5	99.3	> 64	17.5	> 64	64.6	> 32	8.7	> 64	9.0	> 64	38.1	> 8	85.9	> 16	39.2	> 8	0.9	> 8	15.7	> 16	10.3	2	94.6
MBL + (265)	0.5	100	> 64	15.5	> 64	58.9	> 32	0.8	> 64	0.4	> 64	1.9	> 8	85.7	> 16	38.9	> 8	1.1	> 8	11.7	> 16	1.5	2	96.6
KPC + (7)	NA^k^	100	NA	0	NA	85.7	NA	0	NA	0	NA	100	NA	100	NA	28.6	NA	0	NA	0	NA	14.3	NA	85.7
OXA-48-like + (165)	0.5	100	> 64	12.1	> 64	77.0	> 32	12.1	> 64	13.3	1	98.8	2	98.8	> 16	36.4	> 8	12.1	> 8	11.5	> 16	30.3	2	91.5
ESBL + (834)	0.12	99.9	> 64	4.6	8	96.2	> 32	5.3	> 64	9.6	0.5	98.7	1	97.1	> 16	45.8	0.5	96.8	> 8	26.4	≤ 0.06	97.8	1	98.2
Asia																								
All Enterobacterales (8172)	0.25	99.4	> 64	58.8	> 64	86.8	> 32	60.0	> 64	57.0	> 64	88.4	> 8	82.4	> 16	72.5	> 8	73.4	> 8	51.4	> 16	82.8	2	96.3
MDR (4125)	1	98.9	> 64	18.7	> 64	73.8	> 32	21.2	> 64	16.0	> 64	77.1	> 8	83.7	> 16	47.6	> 8	55.6	> 8	16.2	> 16	65.9	2	93.3
XDR (1360)	4	97.7	> 64	5.4	> 64	29.4	> 32	1.0	> 64	0.7	> 64	35.1	> 8	82.7	> 16	15.2	> 8	3.8	> 8	1.5	> 16	8.3	2	90.5
CRE (1419)	2	98.2	> 64	11.0	> 64	35.7	> 32	2.7	> 64	3.2	> 64	35.2	> 8	85.4	> 16	25.1	> 8	0.5	> 8	5.4	> 16	3.5	2	94.6
MBL + (892)	4	97.9	> 64	12.3	> 64	34.5	> 32	0.1	> 64	0.1	> 64	0.6	> 8	85.9	> 16	25.9	> 8	0.8	> 8	5.2	> 16	1.8	2	93.5
KPC + (39)	1	100	> 64	0	> 64	64.1	> 32	0	> 64	0	4	97.4	> 8	69.2	> 16	25.6	> 8	0.0	> 16	0.0	> 16	0.0	4	89.7
OXA-48-like + (416)	0.5	99.5	> 64	4.1	> 64	33.4	> 32	1.9	> 64	3.6	2	96.4	> 8	87.0	> 16	20.7	> 8	7.7	> 8	1.0	> 16	7.0	2	97.4
ESBL + (906)	0.25	98.9	> 64	5.5	32	89.8	> 32	5.0	> 64	13.1	1	98.2	1	95.5	> 16	58.6	1	90.1	> 8	19.8	0.25	92.9	2	96.1
Eurasia																								
All Enterobacterales (2003)	0.25	99.7	> 64	51.6	> 64	86.8	> 32	51.2	> 64	52.2	1	96.4	> 8	81.3	> 16	71.7	8	72.8	> 8	48.1	16	83.7	2	96.9
MDR (1160)	0.5	99.5	> 64	16.6	> 64	77.2	> 32	16.6	> 64	18.8	2	93.8	> 8	79.7	> 16	51.9	> 8	58.9	> 8	19.7	> 16	72.0	2	94.9
XDR (279)	0.5	98.6	> 64	3.2	> 64	29.7	> 32	1.1	> 64	1.8	> 64	76.7	> 8	65.9	> 16	15.1	> 8	3.2	> 8	1.1	> 16	15.8	2	90.3
CRE (323)	0.5	99.1	> 64	12.1	> 64	42.7	> 32	5.0	> 64	7.4	> 64	79.6	> 8	68.7	> 16	38.7	> 8	0.6	> 8	4.0	> 16	4.6	2	95.4
MBL + (67)	0.5	98.5	> 64	14.9	> 64	28.4	> 32	0	> 64	0.0	> 64	4.5	> 8	86.6	> 16	28.4	> 8	3.0	> 8	9.0	> 16	3.0	2	97.0
KPC + (51)	0.5	100	> 64	0	32	70.6	> 32	0	> 64	0.0	4	98.0	8	82.4	> 16	70.6	> 8	0	> 8	2.0	> 16	0	2	100
OXA-48-like + (219)	0.5	99.5	> 64	13.2	> 64	41.6	> 32	6.8	> 64	11.9	2	100	> 8	61.6	> 16	35.2	> 8	2.7	> 8	3.2	> 16	11.9	2	95.4
ESBL + (471)	0.25	99.6	> 64	9.6	16	90.0	> 32	6.6	> 64	19.3	1	98.9	1	94.3	> 16	56.7	1	93.4	> 8	23.6	0.12	97.2	2	97.0
Latin America																								
All Enterobacterales (9517)	0.25	> 99.9	> 64	62.7	8	93.6	> 32	63.4	> 64	63.7	1	95.7	> 8	80.3	> 16	74.2	8	78.6	> 8	58.3	4	88.4	2	96.3
MDR (4331)	0.25	> 99.9	> 64	18.5	32	86.2	> 32	20.3	> 64	21.0	4	90.6	> 8	81.1	> 16	45.3	> 8	63.8	> 8	23.6	> 16	74.5	2	92.9
XDR (907)	0.5	99.9	> 64	7.1	> 64	54.9	> 32	1.0	> 64	5.1	> 64	62.0	> 8	70.7	> 16	16.8	> 8	10.7	> 8	3.5	> 16	13.3	4	86.1
CRE (1101)	0.5	100	> 64	12.4	> 64	64.9	> 32	5.5	> 64	9.6	> 64	64.9	> 8	72.7	> 16	42.5	> 8	1.2	> 8	17.5	> 16	6.0	2	93.8
MBL + (386)	0.5	100	> 64	25.6	> 64	50.8	> 32	0.8	> 64	0.3	> 64	0.8	> 8	75.4	> 16	26.7	> 8	0.5	> 8	18.4	> 16	1.8	2	92.0
KPC + (608)	0.5	100	> 64	0.8	64	69.7	> 32	5.1	> 64	11.7	2	99.2	> 8	69.9	> 16	49.7	> 8	1.8	> 8	14.8	> 16	4.4	2	95.1
OXA-48-like + (31)	0.5	100	> 64	6.5	4	100	> 32	0	> 64	9.7	2	100	1	100	> 16	25.8	> 8	32.3	> 8	6.5	> 16	32.3	2	100
ESBL + (1394)	0.25	100	> 64	5.2	16	93.5	> 32	3.9	> 64	12.7	1	99.6	1	97.1	> 16	42.5	0.5	95.8	> 8	19.8	0.25	93.8	1	97.3

^a^*AMK* amikacin, *ATM* aztreonam, *ATM–AVI* aztreonam–avibactam, *CAZ* ceftazidime, *CZA* ceftazidime-avibactam, *CST* colistin, *FEP* cefepime, *GEN* gentamicin, *IMP* imipenem, *MEM* meropenem, *LVX* levofloxacin, *TGC* tigecycline

^b^Countries were grouped into regions as follows: the Africa/Middle East region included Cameroon, Ivory Coast, Jordan, Kuwait, Morocco, Nigeria, Qatar, Saudi Arabia, and South Africa; Asia included Hong Kong, India, Malaysia, the Philippines, Taiwan, and Thailand; Eurasia included Russia and Turkey; and Latin America included Argentina, Brazil, Chile, Colombia, Costa Rica, Dominican Republic, Guatemala, Mexico, Panama, and Venezuela

^c^For colistin, the CLSI intermediate value is given

^d^For tigecycline, the FDA susceptible breakpoint was used as CLSI does not publish MIC breakpoints for tigecycline

^e^A tentative aztreonam–avibactam pharmacokinetic/pharmacodynamic (PK/PD) susceptible breakpoint of ≤ 8 mg/L was applied for comparative purposes based on recent peer-reviewed publications[9–11]

^f^Isolates were categorized as MDR or XDR according to criteria defined in 2012 by the joint European and US Centers for Disease Control [12], which specify MDR as nonsusceptible to ≥ 1 agent in ≥ 3 antimicrobial classes and XDR as susceptible to ≤ 2 classes. The antimicrobial classes and drug representatives in this analysis included cephalosporins (ceftazidime, cefepime), cephalosporin combined with β-lactamase inhibitors (ceftazidime-avibactam), carbapenems (imipenem, meropenem), fluoroquinolones (levofloxacin), aminoglycosides (gentamicin, amikacin), monobactams (aztreonam), polymyxins (colistin), and glycylcyclines (tigecycline)

^g^Carbapenem-resistant Enterobacterales (CRE) was defined as imipenem or meropenem MIC values of ≥ 4 mg/L. Imipenem was excluded for Morganellaceae due to their intrinsically elevated MIC values

^h^KPC-positive isolates excluded those co-carrying MBLs

^i^OXA-48-like-positive isolates excluded those co-carrying MBLs or KPC

^j^ESBL-positive isolates excluded those carrying carbapenemases

^k^*NA* not applicable (MIC_90_ not calculated when *n* < 10)

Aztreonam–avibactam displayed consistently potent activity against Enterobacterales across all geographic regions analyzed. At a concentration of ≤ 8 mg/L, aztreonam–avibactam was inhibited from 99.4% (Asia) to > 99.9% (Latin America) of isolates in each geographic region (Table 1). Only seven of 5245 isolates from countries in the Africa/Middle East region displayed an aztreonam–avibactam MIC value > 8 mg/L: two from Nigeria, two from Morocco, and one each from Ivory Coast, Saudi Arabia, and Qatar. These organisms included two *E. coli*, and one each of *K. pneumoniae*, *Morganella morganii*,* P. mirabilis*, *Enterobacter asburiae*, and unspeciated *Enterobacter* sp. Among countries in Asia, 45 of 8172 isolates had an aztreonam–avibactam MIC value > 8 mg/L, including *E. coli* (*n* = 31), *K. pneumoniae* (*n* = 5), *P. mirabilis* (*n* = 3), *Providencia rettgeri* (*n* = 3), *Citrobacter koseri* (*n* = 1), *Providencia stuartii* (*n* = 1), and unspeciated *Providencia* sp. (*n* = 1). The great majority of these isolates (41/45, 91.1%) were collected in India, with two originating from Thailand and one each from the Philippines and Taiwan. Only six of 2003 isolates collected in Eurasian countries tested with MIC values > 8 mg/L: five originated from Turkey and one from Russia, consisting of four *E. coli* and one each of *Citrobacter freundii* and *K. pneumoniae*. Just three of the 9517 isolates collected in Latin American countries, one *E. coli*, one *Klebsiella aerogenes* isolate originating from Colombia, and one *P. mirabilis* isolate from Brazil, displayed aztreonam–avibactam MIC values > 8 mg/L.

For the full collection, 12,192 (48.9%), 2974 (11.9%), and 3289 (13.2%) of isolates were identified as MDR, XDR, and CRE, respectively. Aztreonam–avibactam retained in vitro activity against these phenotypic subsets of Enterobacterales isolates, with 99.5% of MDR, 98.7% of XDR, and 99.1% of CRE isolates inhibited at ≤ 8 mg/L (Table 1). No comparator agent inhibited > 90% of MDR, XDR, or CRE isolates other than tigecycline which inhibited 93.4% of MDR and 94.4% of CRE isolates.

**Fig. 1:**
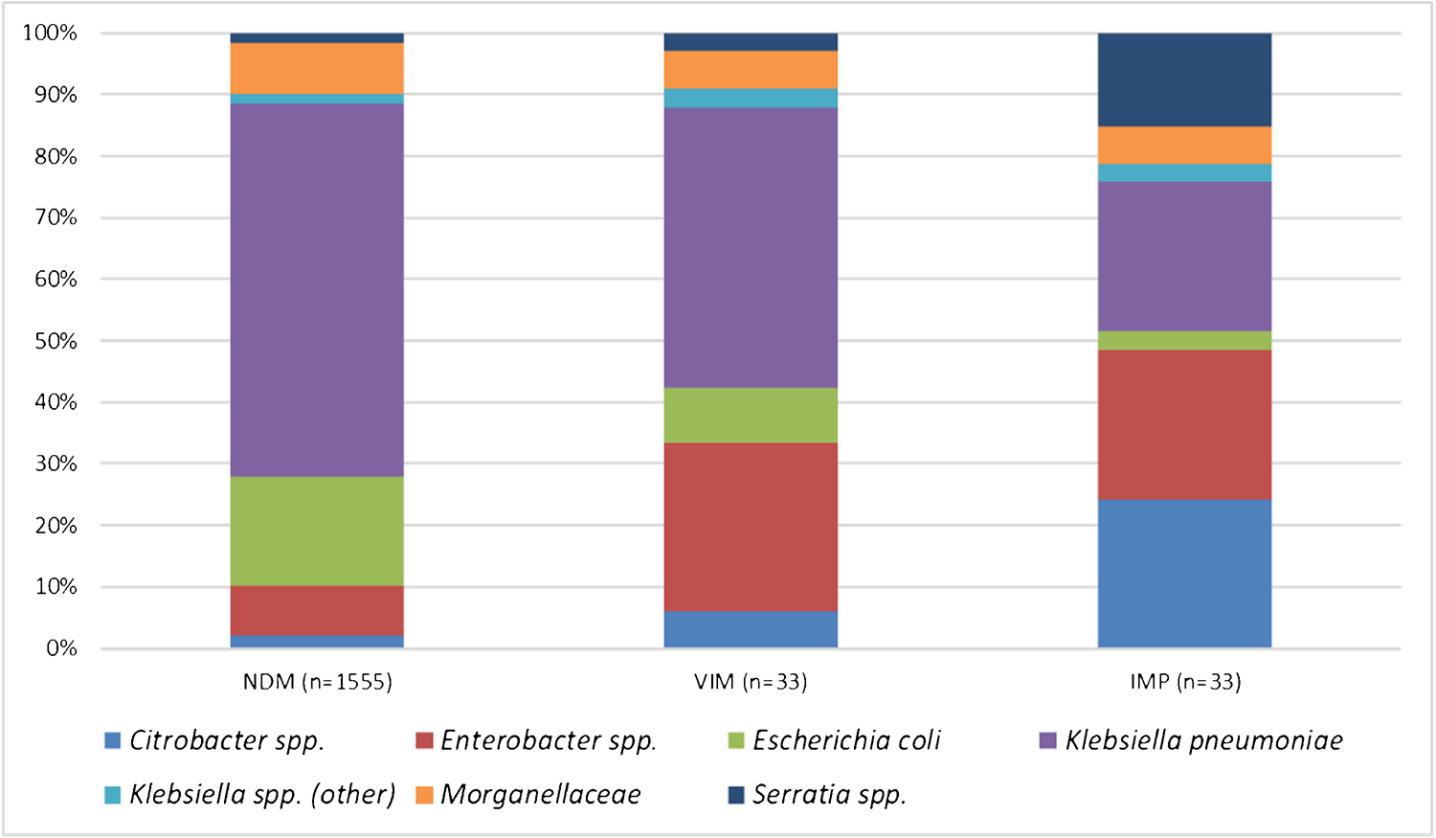
Taxonomic classification of MBL-positive Enterobacterales by MBL family. Isolates that co-carried NDM and VIM (*n* = 6) and NDM and IMP (*n* = 5) are counted for each enzyme family category. Poorly visible: one *Raoultella* sp. carrying NDM

Overall, 7446 Enterobacterales isolates were eligible for β-lactamase gene screening based upon the qualifying criteria. Among these, 1610 were identified as carrying one or more MBLs, including 1544 isolates carrying NDM, 27 carrying VIM, 28 carrying IMP, six co-carrying NDM and VIM, and five co-carrying NDM and IMP. Figure 1 provides the taxonomic distribution of MBL-producing isolates by enzyme family. *K. pneumoniae* harbored NDM and VIM more frequently (60.1% and 45.4% of the NDM and VIM carriers, respectively) than other species of Enterobacterales, whereas IMP was found in an equal number of *K. pneumoniae* and *Enterobacter* spp. isolates (each genus/species accounted for 24.2% of all IMP carriers). Aztreonam–avibactam at a concentration of ≤ 8 mg/L (MIC_90_, 2 mg/L) inhibited 98.8% of all MBL producers, including 100% of VIM and IMP carriers and 98.7% of NDM carriers. All comparator antimicrobial agents were inactive against MBL producers with the exception of tigecycline which inhibited 93.8% of isolates (Table 1). It should be noted that the meropenem-nonsusceptibility threshold that triggered screening (MIC of ≥ 2 mg/L) could potentially miss some carbapenemase-carrying organisms for which the carbapenemase is weakly expressed [14].

In addition, among the molecularly characterized isolates, 705 were identified that harbored KPC (excluding those that co-carried an MBL), 831 carried an OXA-48-like enzyme (excluding those that co-carried an MBL or KPC), and 3605 harbored an ESBL (excluding those that co-carried a carbapenemase). Aztreonam–avibactam at a concentration of ≤ 8 mg/L inhibited 100%, 99.6%, and 99.6% of KPC-, OXA-48-like-, and ESBL-carrying isolates, respectively (Table 1). Among comparator agents, > 98% of KPC-, OXA-48-like-, and ESBL-carrying isolates were ceftazidime–avibactam-susceptible and ≥ 95% were tigecycline-susceptible.

**Fig. 2:**
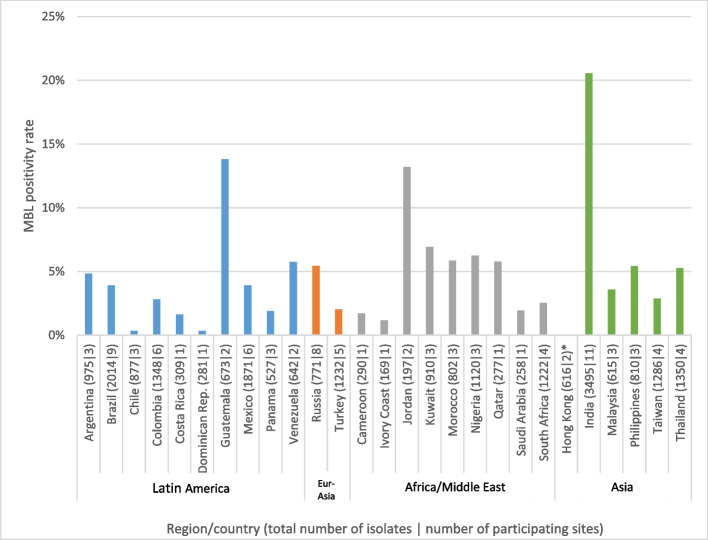
Incidence of MBL positivity among isolates collected for the ATLAS surveillance project in 2019–2021 by country. Asterisk indicates no MBL producers collected in Hong Kong

**Fig. 3:**
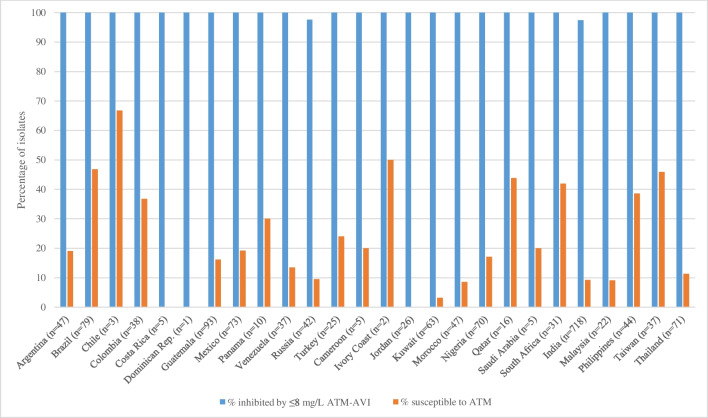
Percentages of MBL producers (*n* = 1610) inhibited by aztreonam–avibactam (ATM–AVI) at a concentration of ≤ 8 mg/L and susceptible to aztreonam (ATM) alone (MIC ≤ 4 mg/L) by country

Given the potential for aztreonam–avibactam to treat patients infected with MBL-producing Enterobacterales isolates, further analyses were performed on the subset of 1610 isolates that carried MBLs, the great majority of which harbored genes for NDM-type enzymes (1555/1610, 96.6% carried NDM, either as the sole MBL or with additional MBLs). Figure 2 summarizes the percentage of MBL producers identified among the total number of isolates collected in each of the evaluated countries. MBL-positive isolates were most common in India (20.5%), Guatemala (13.8%), and Jordan (13.2%), three geographically diffuse countries, but were rare (≤ 2%) in Cameroon, Chile, Costa Rica, Dominican Republic, Ivory Coast, Panama, Saudi Arabia, and Turkey, and completely absent in Hong Kong. Aztreonam–avibactam at a concentration of ≤ 8 mg/L inhibited 100%, 100%, 98.5%, and 97.9%, of MBL-positive Enterobacterales isolates from the Africa/Middle Eastern, Latin American, Eurasian, and Asian regions, respectively (Table 1). Figure 3 provides country-specific data for both aztreonam–avibactam and aztreonam against MBL-harboring isolates and shows that at a concentration of ≤ 8 mg/L, aztreonam–avibactam inhibited the growth of all MBL-harboring isolates from all countries but two: India, where aztreonam–avibactam at a concentration of ≤ 8 mg/L inhibited 97.4% of isolates, and Russia, where 97.6% of isolates were inhibited. These results are in contrast to aztreonam alone, against which the susceptibility of MBL-harboring isolates ranged from 66.7% in Chile to 0% in Costa Rica, Dominican Republic, and Jordan.

## Discussion

Aztreonam–avibactam demonstrated potent activity against a recent set of Enterobacterales originating from twenty-seven countries in Latin America, Eurasia, Africa/Middle East, and Asia. Importantly, the in vitro activity of aztreonam–avibactam was maintained against MBL producers, a group that currently presents clinicians with limited treatment options. The results presented here on contemporary isolates (2019–2021) confirm earlier reports on global collections focused on aztreonam–avibactam susceptibility. Karlowsky et al. described the activity of aztreonam–avibactam and comparators against a large dataset including Enterobacterales (*n* = 51,352) and *Pseudomonas aeruginosa* (*n* = 11,842) collected from 40 countries from 2012 to 2015 [15]. That study reported that > 99.9% of all Enterobacterales isolates and 99.8% of meropenem-nonsusceptible Enterobacterales (*n* = 1498) were inhibited by aztreonam–avibactam at a concentration of ≤ 8 mg/L. Additionally, all 267 Enterobacterales isolates positive for MBL genes (NDM, VIM, IMP) demonstrated aztreonam–avibactam MICs of ≤ 8 mg/L. An earlier study by Biedenbach et al. evaluated aztreonam–avibactam and comparator antimicrobial agents against 28,501 unique clinical isolates of Enterobacterales, *P. aeruginosa*, and *Acinetobacter baumannii* collected in 39 countries in 2012–2013 [16]. In that study, aztreonam–avibactam inhibited 99.9% of all Enterobacterales, and > 99% of the MBL-producing isolates of Enterobacterales at ≤ 4 mg/L. Sader et al. recently reported on a set of 24,924 Enterobacterales collected in Europe, Asia, and Latin America as a part of the SENTRY program from both developed and developing countries in 2019–2021 [17]. They demonstrated that 99.6% of CRE were inhibited at an aztreonam–avibactam concentration of ≤ 8 mg/L, including 99.9% of isolates carrying a carbapenemase gene. Similarly, Rossolini analyzed Enterobacterales isolates collected globally (54 countries) in 2019 and showed that aztreonam–avibactam possessed potent antimicrobial activity against all Enterobacterales, with 99.9% of isolates, including > 99% of those carrying MBLs, inhibited at a concentration of ≤ 8 mg/L [18].

In the present study, only 61 of 24,937 (0.2%) isolates were tested with an aztreonam–avibactam MIC > 8 mg/L, including 38 *E. coli*, seven *K. pneumoniae*, five *P. mirabilis*, three *Providencia rettgeri*, and eight members of other species of Enterobacterales. In *E. coli*, elevated aztreonam–avibactam MIC values have been associated with four amino acid insertions in a region of the penicillin-binding protein 3 (PBP3) adjacent to the β-lactam binding pocket [19–21]. Recently, Sadek et al. demonstrated that although the previously described PBP3 insertions contribute to decreased aztreonam–avibactam susceptibility, they cannot be considered the unique basis of resistance as the presence of a transmissible AmpC-type β-lactamase, CMY (particularly the CMY-42 variant), and also contributes to elevated MICs [22]. Although a thorough investigation of mechanisms of resistance to aztreonam–avibactam was beyond the scope of this report, it is noteworthy that 28 of the 38 (71.1%) *E. coli* isolates with aztreonam–avibactam MIC values > 8 mg/L were found to harbor CMY (Supplemental Table S3). Twenty of those carried CMY-42, six carried CMY-145, and one each carried CMY-141 and CMY-146. CMY-141, CMY-145, and CMY-146 each differ from CMY-42 by a single amino acid substitution. Additionally, recent evidence suggests that an elevation of CMY gene expression levels, as would be expected with an increase in the copy number of the plasmid carrying the gene, also contributes to reduced aztreonam–avibactam susceptibility [23].

In conclusion, the current data on recent clinical isolates collected worldwide confirms the potent activity of aztreonam–avibactam against Enterobacterales, including isolates that carry MBLs. As MBL-producing Enterobacterales become more prevalent in healthcare systems across regions and therapeutic options to treat serious infections caused by MBL carriers are presently extremely limited, aztreonam–avibactam represents a potentially valuable option for the treatment of infections caused by MBL-producing MDR and XDR Enterobacterales.

## Supplementary Information

Below is the link to the electronic supplementary material.

Supplementary file1 (DOCX 38 KB)

## Data Availability

The data generated and/or analyzed for the current study are available from the corresponding author on reasonable request.
