# Supplementary material for: In vitro activity of aztreonam–avibactam against Enterobacterales isolates collected in Latin America, Africa/Middle East, Asia, and Eurasia for the ATLAS Global Surveillance Program in 2019–2021
Source: Eur J Clin Microbiol Infect Dis. 2023 Aug 1;42(9):1135–43. doi: 10.1007/s10096-023-04645-2 (PMC10427541; doi:10.1007/s10096-023-04645-2)
Supplement: Supplementary file 1 — Supplementary file1 (DOCX 38 KB) [file 10096_2023_4645_MOESM1_ESM.docx]

## Supplemental Table S1

## Isolates characteristics of the 27937 Enterobacterales^a^ isolates tested in this study.

| **Parameter** | **Parameter subset** | **No. of isolates with parameter** |
| --- | --- | --- |
| Infection source | Bloodstream | 5921 |
|  | Intra-abdominal | 3263 |
|  | Lower respiratory tract | 4907 |
|  | Skin and soft tissue | 4400 |
|  | Urinary tract | 6408 |
|  | Other/Not-specified | 38 |
|  |  |  |
| Country of isolation | Argentina | 975 |
|  | Brazil | 2014 |
|  | Cameroon | 290 |
|  | Chile | 877 |
|  | Colombia | 1348 |
|  | Costa Rica | 309 |
|  | Dominican Republic | 169 |
|  | Guatemala | 281 |
|  | Hong Kong | 673 |
|  | India | 616 |
|  | Ivory Coast | 3495 |
|  | Jordan | 197 |
|  | Kuwait | 910 |
|  | Malaysia | 615 |
|  | Mexico | 1871 |
|  | Morocco | 802 |
|  | Nigeria | 1120 |
|  | Panama | 527 |
|  | Philippines | 810 |
|  | Qatar | 277 |
|  | Russia | 771 |
|  | Saudi Arabia | 258 |
|  | South Africa | 1222 |
|  | Taiwan | 1286 |
|  | Thailand | 1350 |
|  | Turkey | 1232 |
|  | Venezuela | 642 |
|  |  |  |
| Hospital ward type | ICU | 6632 |
|  | non-ICU | 17852 |
|  | not specified | 453 |
|  |  |  |
| Patient age | Pediatric (<18) | 3102 |
|  | Adult (18-64) | 12945 |
|  | Elderly (>64) | 8890 |
|  |  |  |
| Project year | 2019 | 8056 |
|  | 2020 | 8689 |
|  | 2021 | 8192 |

^a^Includes *Citrobacter* *amalonaticus* (*n*=25), *Citrobacter* *braakii* (*n*=34), *Citrobacter* *farmer* (*n*=5), *Citrobacter* *freundii* (*n*=456), *Citrobacter* *freundii* complex (*n*=2), *Citrobacter gillenii* (n=1), *Citrobacter* *koseri* (*n*=466), *Citrobacter* *sedlakii* (*n*=14), *Citrobacter* sp. (*n*=41), *Enterobacter* *asburiae* (*n*=96), *Enterobacter* *bugandensis* (*n*=240), *Enterobacter* *cloacae* (*n*=1225), *Enterobacter* *cloacae* complex (*n*=112), *Enterobacter* *hormaechi*  (n=1), *Enterobacter kobei* (*n*=13), *Enterobacter* *ludwigii* (*n*=4), *Enterobacter* sp. (*n*=720), *Enterobacter* *xiangfangensis* (*n*=78), *Escherichia* *coli* (*n*=7844), *Klebsiella* *aerogenes* (*n*=428), *Klebsiella* *oxytoca* (*n*=414), *Klebsiella* *pneumoniae* (*n*=8627), *Klebsiella* sp. (*n*=48), *Klebsiella* *variicola* (*n*=271), *Morganella* *morganii* (*n*=723), *Pantoea* *agglomerans* (*n*=1), *Pluralibacter* *gergoviae* (*n*=1), *Proteus* *hauseri* (*n*=53), *Proteus* *mirabilis* (*n*=1052), *Proteus* *penneri* (*n*=7), *Proteus* sp. (*n*=65), *Proteus* *vulgaris* (*n*=130), *Providencia* *alcalifaciens* (*n*=5), *Providencia* *rettgeri* (*n*=217), *Providencia* sp. (*n*=36), *Providencia* *stuartii* (*n*=243), *Raoultella* *ornithinolytica* (*n*=8), *Raoultella* *planticola* (*n*=1), *Raoultella* sp. (*n*=1), *Salmonella* sp. (*n*=5), *Serratia* *liquefaciens* (*n*=5), *Serratia* *marcescens* (*n*=1115), *Serratia* *rubidaea* (*n*=1), *Serratia* sp. (*n*=100), and *Serratia* *ureilytica* (*n*=3).

## Supplemental Table S2

### *In vitro* activity of aztreonam-avibactam and comparators agents against Enterobacterales collected in emerging market countries, 2019-2021, interpreted with 2022 EUCAST MIC breakpoints [7].

| Region^b^  Phenotype/genotype (no. of isolates) | Antimicrobial agent^a^  % susceptible (%S) | | | | | | | | | | | |
| --- | --- | --- | --- | --- | --- | --- | --- | --- | --- | --- | --- | --- |
|  | ATM-AVI | ATM | AMK | FEP | CAZ | CZA | CST | GEN | IPM | LVX | MEM | TGC^c^ |
|  | % ≤8 mg/L^d^ | %S | %S | %S | %S | %S | %S | %S | %S | %S | %S | %S |
| All regions |  | | | | | | | | | | | |
| All Enterobacterales (24,937) | 99.8 | 56.6 | 88.4 | 58.6 | 55.1 | 93.1 | 81.5 | 71.6 | 83.8 | 54.8 | 87.8 | NA |
| MDR^e^ (12,192) | 99.5 | 14.4 | 77.0 | 17.2 | 12.4 | 86.0 | 82.7 | 44.4 | 70.1 | 20.8 | 75.2 | NA |
| XDR^e^ (2,974) | 98.7 | 4.7 | 35.2 | 0.8 | 0.8 | 47.6 | 77.2 | 16.1 | 14.0 | 2.7 | 17.0 | NA |
| CRE^f^ (3,289) | 99.1 | 10.4 | 42.5 | 3.8 | 4.0 | 49.9 | 79.6 | 33.0 | 2.3 | 10.7 | 7.8 | NA |
| MBL+ (1610) | 98.8 | 13.2 | 33.4 | 0.2 | 0.0 | 1.0 | 83.4 | 27.0 | 2.0 | 9.6 | 3.4 | NA |
| KPC+^g^ (705) | 100 | 0.0 | 60.4 | 2.4 | 3.0 | 99.0 | 71.1 | 49.1 | 3.5 | 13.6 | 8.4 | NA |
| OXA-48-like+^h^ (831) | 99.6 | 7.2 | 42.6 | 3.1 | 4.9 | 98.0 | 81.7 | 26.6 | 19.0 | 3.9 | 21.7 | NA |
| ESBL+^i^ (3,605) | 99.6 | 0.4 | 87.6 | 2.1 | 3.8 | 98.9 | 96.3 | 47.9 | 96.9 | 21.8 | 97.4 | NA |
| Africa/Middle East |  | | | | | | | | | | | |
| All Enterobacterales (5,245) | 99.9 | 56.5 | 92.3 | 57.7 | 54.8 | 94.5 | 82.5 | 69.4 | 88.6 | 56.2 | 92.7 | NA |
| MDR (2,576) | 99.7 | 14.8 | 85.3 | 15.8 | 12.6 | 88.9 | 85.2 | 39.9 | 80.4 | 24.2 | 85.2 | NA |
| XDR (428) | 98.8 | 4.7 | 51.6 | 1.6 | 0.5 | 37.9 | 80.8 | 22.7 | 18.9 | 5.8 | 23.8 | NA |
| CRE (446) | 99.3 | 15.9 | 52.5 | 7.4 | 7.4 | 38.1 | 85.9 | 38.1 | 3.6 | 15.7 | 14.6 | NA |
| MBL+ (265) | 100 | 12.8 | 42.3 | 0.8 | 0.0 | 1.9 | 85.7 | 37.4 | 4.2 | 11.7 | 4.2 | NA |
| KPC+ (7) | 100 | 0.0 | 85.7 | 0.0 | 0.0 | 100.0 | 100.0 | 28.6 | 0.0 | 0.0 | 14.3 | NA |
| OXA-48-like+ (165) | 100 | 11.5 | 70.3 | 7.3 | 7.9 | 98.8 | 91.5 | 35.8 | 33.9 | 11.5 | 43.6 | NA |
| ESBL+ (834) | 99.9 | 0.0 | 91.2 | 1.9 | 2.2 | 98.7 | 97.1 | 44.5 | 97.7 | 26.4 | 98.3 | NA |
| Asia |  | | | | | | | | | | | |
| All Enterobacterales (8,172) | 99.4 | 55.4 | 84.7 | 58.4 | 52.7 | 88.4 | 82.4 | 71.4 | 79.9 | 51.4 | 83.4 | NA |
| MDR (4,125) | 98.9 | 14.6 | 70.3 | 18.9 | 10.7 | 77.1 | 83.7 | 46.1 | 63.0 | 16.2 | 67.2 | NA |
| XDR (1,360) | 97.7 | 4.3 | 25.4 | 0.7 | 0.2 | 35.1 | 82.7 | 14.7 | 10.1 | 1.5 | 11.0 | NA |
| CRE (1,419) | 98.2 | 8.9 | 31.6 | 2.4 | 2.2 | 35.2 | 85.4 | 24.4 | 1.9 | 5.4 | 4.5 | NA |
| MBL+ (892) | 97.9 | 10.1 | 28.9 | 0.1 | 0.0 | 0.6 | 85.9 | 25.2 | 1.6 | 5.2 | 2.8 | NA |
| KPC+ (39) | 100 | 0.0 | 64.1 | 0.0 | 0.0 | 97.4 | 69.2 | 25.6 | 0.0 | 12.8 | 0.0 | NA |
| OXA-48-like+ (416) | 99.5 | 3.8 | 32.0 | 1.2 | 2.2 | 96.4 | 87.0 | 19.2 | 12.7 | 1.0 | 11.5 | NA |
| ESBL+ (906) | 98.9 | 0.8 | 86.1 | 2.1 | 3.3 | 98.2 | 95.5 | 56.7 | 94.6 | 19.8 | 95.1 | NA |
| Eurasia |  |  |  |  |  |  |  |  |  |  |  |  |
| All Enterobacterales (2,003) | 99.7 | 46.8 | 82.3 | 48.9 | 46.6 | 96.4 | 81.3 | 71.0 | 80.6 | 48.1 | 85.1 | NA |
| MDR (1,160) | 99.5 | 11.8 | 69.8 | 13.9 | 11.8 | 93.8 | 79.7 | 51.1 | 68.3 | 19.7 | 74.5 | NA |
| XDR (279) | 98.6 | 2.2 | 22.9 | 1.1 | 0.7 | 76.7 | 65.9 | 14.0 | 15.1 | 1.1 | 21.9 | NA |
| CRE (323) | 99.1 | 9.6 | 33.1 | 3.4 | 5.3 | 79.6 | 68.7 | 37.8 | 3.1 | 4.0 | 7.7 | NA |
| MBL+ (67) | 98.5 | 11.9 | 11.9 | 0.0 | 0.0 | 4.5 | 86.6 | 28.4 | 3.0 | 9.0 | 4.5 | NA |
| KPC+ (51) | 100 | 0.0 | 68.6 | 0.0 | 0.0 | 98.0 | 82.4 | 70.6 | 0.0 | 2.0 | 0.0 | NA |
| OXA-48-like+ (219) | 99.5 | 10.5 | 33.8 | 4.1 | 7.8 | 100.0 | 61.6 | 34.2 | 17.4 | 3.2 | 22.4 | NA |
| ESBL+ (471) | 99.6 | 0.8 | 81.1 | 3.4 | 7.0 | 98.9 | 94.3 | 55.8 | 96.0 | 23.6 | 98.3 | NA |
| Latin America |  |  |  |  |  |  |  |  |  |  |  |  |
| All Enterobacterales (9,517) | >99.9 | 59.8 | 90.6 | 61.4 | 59.3 | 95.7 | 80.3 | 73.2 | 85.1 | 58.3 | 89.5 | NA |
| MDR (4,331) | >99.9 | 14.7 | 80.5 | 17.2 | 14.1 | 90.6 | 81.1 | 43.8 | 71.3 | 23.6 | 77.0 | NA |
| XDR (907) | 99.9 | 6.1 | 45.8 | 0.6 | 2.0 | 62.0 | 70.7 | 15.7 | 17.2 | 3.5 | 21.4 | NA |
| CRE (1,101) | 100 | 10.3 | 55.2 | 4.2 | 4.7 | 64.9 | 72.7 | 40.6 | 2.2 | 17.5 | 9.3 | NA |
| MBL+ (386) | 100 | 21.0 | 41.5 | 0.0 | 0.0 | 0.8 | 75.4 | 23.6 | 1.6 | 18.4 | 3.9 | NA |
| KPC+ (608) | 100 | 0.0 | 59.2 | 2.8 | 3.5 | 99.2 | 69.9 | 49.0 | 4.1 | 14.8 | 9.5 | NA |
| OXA-48-like+ (31) | 100 | 6.5 | 100.0 | 0.0 | 6.5 | 100.0 | 100.0 | 22.6 | 35.5 | 6.5 | 35.5 | NA |
| ESBL+ (1,394) | 100 | 0.4 | 88.5 | 1.9 | 4.0 | 99.6 | 97.1 | 41.6 | 98.3 | 19.8 | 97.9 | NA |

^a^ Abbreviations: AMK, amikacin; ATM, aztreonam; ATM-AVI, aztreonam-avibactam; CAZ, ceftazidime; CZA, ceftazidime-avibactam, CST, colistin; FEP, cefepime; GEN, gentamicin, IMP, imipenem, MEM, meropenem; LVX, levofloxacin; TGC, tigecycline.

^b^ Countries were grouped into regions as follows: the Africa/Middle East region included Cameroon, Ivory Coast, Jordan, Kuwait, Morocco, Nigeria, Qatar, Saudi Arabia, and South Africa; Asia included Hong Kong, India, Malaysia, Philippines, Taiwan, and Thailand; Eurasia included Russia and Turkey; and Latin America included Argentina, Brazil, Chile, Colombia, Costa Rica, Dominican Republic, Guatemala, Mexico, Panama, and Venezuela.

^c^ For tigecycline, EUCAST publishes breakpoints for *E. coli* and *C. koseri* only.

^d^ A tentative aztreonam-avibactam pharmacokinetic/pharmacodynamic (PK/PD) susceptible breakpoint of ≤8 mg/L was applied for comparative purposes based on recent peer-reviewed publications [9-11].

^e^ Isolates were categorized as MDR or XDR according to criteria defined in 2012 by the joint European and US Centers for Disease Control [12], which specify MDR as nonsusceptible to ≥1 agent in ≥ 3 antimicrobial classes and XDR as susceptible to ≤2 classes. The antimicrobial classes and drug representatives in this analysis included cephalosporins (ceftazidime, cefepime), cephalosporin combined with β-lactamase inhibitors (ceftazidime-avibactam), carbapenems (imipenem, meropenem), fluoroquinolones (levofloxacin), aminoglycosides (gentamicin, amikacin), monobactams (aztreonam), polymyxins (colistin) and glycylcyclines (tigecycline).

^f^ Carbapenem-resistant Enterobacterales (CRE) was defined as imipenem or meropenem MIC values of ≥4 mg/L. Imipenem was excluded for *Morganellaceae* due to their intrinsically elevated MIC values.

^g^ KPC-positive isolates excluded those co-carrying MBLs.

^h^ OXA-48-like-positive isolates excluded those co-carrying MBLs or KPC.

^i^ ESBL-positive isolates excluded those carrying carbapenemases.

## Supplemental Table S3

Characteristics of isolates testing with aztreonam-avibactam MIC values ≥16 mg/L.

| Year | Organism | Country | ATM-AVI^a^ MIC (mg/L) | Acquired β-lactamase summary^b,c^ |
| --- | --- | --- | --- | --- |
| 2019 | *Escherichia coli* | India | 32 | CMY-42 |
| 2019 | *Escherichia coli* | India | 16 | CMY-42; NDM-5 |
| 2019 | *Escherichia coli* | India | 16 | not characterized |
| 2019 | *Escherichia coli* | India | 16 | CMY-42 |
| 2019 | *Escherichia coli* | India | 16 | CMY-42; CTX-M-15 |
| 2019 | *Escherichia coli* | India | 16 | CMY-42; CTX-M-15; TEM-OSBL |
| 2019 | *Escherichia coli* | Russia | 16 | CMY-145; CTX-M-55; NDM-5; TEM-OSBL |
| 2019 | *Escherichia coli* | Turkey | 16 | CMY-42; CTX-M-15; OXA-48 |
| 2019 | *Klebsiella pneumoniae* | Thailand | 16 | DHA-1; SHV-OSBL; TEM-OSBL |
| 2019 | *Proteus mirabilis* | Brazil | >128 | TEM-OSBL |
| 2020 | *Escherichia coli* | India | 16 | CMY-141 |
| 2020 | *Escherichia coli* | India | 16 | CMY-146; CTX-M-15 |
| 2020 | *Escherichia coli* | India | 16 | CTX-M-15; NDM-5; TEM-OSBL |
| 2020 | *Escherichia coli* | India | 32 | CMY-42; NDM-5; TEM-OSBL |
| 2020 | *Escherichia coli* | India | >64 | CMY-145; NDM-5 |
| 2020 | *Escherichia coli* | India | 16 | CMY-42; CTX-M-15 |
| 2020 | *Escherichia coli* | India | 16 | CTX-M-15 |
| 2020 | *Escherichia coli* | India | 32 | CMY-42 |
| 2020 | *Escherichia coli* | India | 16 | CMY-42 |
| 2020 | *Escherichia coli* | India | 16 | CMY-145; TEM-OSBL |
| 2020 | *Escherichia coli* | India | 16 | DHA-1; CTX-M-15; NDM-5; TEM-OSBL |
| 2020 | *Escherichia coli* | India | 16 | CMY-42; CTX-M-15; NDM-5; TEM-OSBL |
| 2020 | *Escherichia coli* | Qatar | 16 | CMY-42 |
| 2020 | *Escherichia coli* | Saudi Arabia | 64 | CMY-42 |
| 2020 | *Klebsiella pneumoniae* | India | >64 | CTX-M-15; NDM-5; SHV-OSBL; TEM-OSBL; OXA-232 |
| 2020 | *Proteus mirabilis* | India | 16 | DHA-TRUNC; NDM-1; TEM-OSBL |
| 2020 | *Proteus mirabilis* | India | 16 | DHA-TRUNC; NDM-1; TEM-OSBL |
| 2020 | *Proteus mirabilis* | Nigeria | >64 | TEM-OSBL |
| 2020 | *Providencia rettgeri* | India | 16 | CMY-4; VEB-9; NDM-1; TEM-OSBL; OXA-48 |
| 2020 | *Providencia sp* | India | 16 | DHA-TRUNC; VEB-24; NDM-1 |
| 2021 | *Citrobacter freundii* | Turkey | 32 | none detected |
| 2021 | *Citrobacter koseri* | Philippines | 32 | none detected |
| 2021 | *Enterobacter asburiae* | Côte d’ Ivoire | >64 | none detected |
| 2021 | *Enterobacter sp* | Morocco | 64 | none detected |
| 2021 | *Escherichia coli* | Colombia | >64 | TEM-OSBL |
| 2021 | *Escherichia coli* | India | 32 | CMY-42 |
| 2021 | *Escherichia coli* | India | >64 | NDM-1 |
| 2021 | *Escherichia coli* | India | >64 | CTX-M-15; TEM-OSBL |
| 2021 | *Escherichia coli* | India | 16 | CMY-42; CTX-M-15 |
| 2021 | *Escherichia coli* | India | 16 | CMY-42; NDM-5 |
| 2021 | *Escherichia coli* | India | 16 | CMY-42; NDM-5; TEM-OSBL |
| 2021 | *Escherichia coli* | India | 16 | not characterized |
| 2021 | *Escherichia coli* | India | 32 | CMY-145; NDM-5; TEM-OSBL |
| 2021 | *Escherichia coli* | India | 16 | CMY-145; NDM-1 |
| 2021 | *Escherichia coli* | India | 16 | CMY-42; CTX-M-15 |
| 2021 | *Escherichia coli* | India | 16 | CMY-145; NDM-1 |
| 2021 | *Escherichia coli* | Taiwan | 32 | CMY-42 |
| 2021 | *Escherichia coli* | Thailand | 16 | TEM-OSBL |
| 2021 | *Escherichia coli* | Turkey | 16 | CTX-M-15 |
| 2021 | *Escherichia coli* | Turkey | 16 | CMY-42; CTX-M-15 |
| 2021 | *Klebsiella aerogenes* | Colombia | 32 | none detected |
| 2021 | *Klebsiella pneumoniae* | India | 16 | CTX-M-15; SHV-OSBL; TEM-OSBL; OXA-232 |
| 2021 | *Klebsiella pneumoniae* | India | >64 | TEM-OSBL |
| 2021 | *Klebsiella pneumoniae* | India | >64 | CTX-M-15; SHV-ESBL; NDM-5; TEM-OSBL; OXA-181 |
| 2021 | *Klebsiella pneumoniae* | Nigeria | >64 | PER-7 |
| 2021 | *Klebsiella pneumoniae* | Turkey | 32 | none detected |
| 2021 | *Morganella morganii* | Morocco | 32 | DHA-1 |
| 2021 | *Proteus mirabilis* | India | 32 | VEB-24; TEM-OSBL |
| 2021 | *Providencia rettgeri* | India | >64 | CMY-4; OXA-181 |
| 2021 | *Providencia rettgeri* | India | >64 | VEB-14; NDM-1 |
| 2021 | *Providencia stuartii* | India | 16 | CMY-4; VEB-24 |

^a^ ATM-AVI, aztreonam-avibactam.

^b^ OSBL, original-spectrum β-lactamase (e.g., TEM-1, SHV-1, etc.).

^c^ TRUNC, truncated.
